# Enhanced non-volatile memory characteristics with quattro-layer graphene nanoplatelets vs*.* 2.85-nm Si nanoparticles with asymmetric Al_2_O_3_/HfO_2_ tunnel oxide

**DOI:** 10.1186/s11671-015-0957-5

**Published:** 2015-06-02

**Authors:** Nazek El-Atab, Berk Berkan Turgut, Ali K Okyay, Munir Nayfeh, Ammar Nayfeh

**Affiliations:** Institute Center for Microsystems—iMicro, EECS, Masdar Institute of Science and Technology Abu Dhabi, Abu Dhabi, United Arab Emirates; UNAM-National Nanotechnology Research Center, Ankara, Turkey; Institute of Material Science and Nanotechnology, Ankara, Turkey; Department of Electrical and Electronics Engineering, Bilkent University, 06800 Ankara, Turkey; Department of Physics, University of Illinois at Urbana Champaign, 1206 W. Green Street, Urbana, IL 61801 USA

**Keywords:** Charge trapping memory devices, Graphene nanoplatelets, Silicon nanoparticles, Aluminum oxide, Atomic layer deposition, Retention time, Program time

## Abstract

In this work, we demonstrate a non-volatile metal-oxide semiconductor (MOS) memory with Quattro-layer graphene nanoplatelets as charge storage layer with asymmetric Al_2_O_3_/HfO_2_ tunnel oxide and we compare it to the same memory structure with 2.85-nm Si nanoparticles charge trapping layer. The results show that graphene nanoplatelets with Al_2_O_3_/HfO_2_ tunnel oxide allow for larger memory windows at the same operating voltages, enhanced retention, and endurance characteristics. The measurements are further confirmed by plotting the energy band diagram of the structures, calculating the quantum tunneling probabilities, and analyzing the charge transport mechanism. Also, the required program time of the memory with ultra-thin asymmetric Al_2_O_3_/HfO_2_ tunnel oxide with graphene nanoplatelets storage layer is calculated under Fowler-Nordheim tunneling regime and found to be 4.1 ns making it the fastest fully programmed MOS memory due to the observed pure electrons storage in the graphene nanoplatelets. With Si nanoparticles, however, the program time is larger due to the mixed charge storage. The results confirm that band-engineering of both tunnel oxide and charge trapping layer is required to enhance the current non-volatile memory characteristics.

**Electronic supplementary material:**

The online version of this article (doi:10.1186/s11671-015-0957-5) contains supplementary material, which is available to authorized users.

## Background

The demand for low-power, high-speed, and high-density non-volatile memory devices has increased drastically over the past decade due to the growing market of consumer electronics. However, current flash memory devices are expected to face two major challenges in the near future: density and voltage scaling. The density of the memory is related to the gate length scaling which is constrained by the gate stack, precisely, the tunnel oxide thickness. In fact, the gate length is required to be adequate with the gate stack in order to maintain a good gate control and to avoid short channel effects. However, in conventional flash memories, the tunnel oxide thickness has a lower limit of 6–8 nm (depending on NOR or NAND structure) in order to avoid back-tunneling and thus leakage of charges which destroys the necessary retention characteristic of the memory (>10 years). The second problem which needs to be solved is the high program and erase operating voltages. Once again, the limitation to operating voltage scaling is the inability to reduce gate stack thickness. In addition to the trade-off relationship between tunnel oxide thickness and retention characteristic of the memory where the retention of charges is exponentially degraded as the tunnel oxide thickness is scaled down, there exists another trade-off relationship between the tunnel oxide thickness and the resulting program time, where a thicker tunnel oxide causes the extension of the time needed for the charges to be transported from the channel to the charge trapping layer and vice-versa. Therefore, it is imperative to find novel structures and materials to be incorporated in the memory cells which would allow tunnel oxide and voltage scaling.

Since its first discovery in 2004 [1], graphene has attracted major attention and is currently considered as a promising material in next-generation information-processing devices due to its outstanding electronic properties [2]. However, the sole use of pristine graphene as the charge storage layer is not enough to enhance the current non-volatile memory characteristics [3]. The choice of the tunnel oxide material of the memory has a significant impact on the memory performance [4]. On the other hand, Si-nanoparticle-based memory has been extensively investigated, and on the industry side, it was considered as a viable memory system due to the larger retention time, lower power consumption, and faster operation than conventional polysilicon-based flash memory [5, 6]. Freescale demonstrated a 4-Mbit flash memory device as early as 2003 and has most recently (2006) demonstrated a 24-Mbit flash memory device using Si nanoparticle materials.

In this work, we demonstrate a non-volatile metal-oxide semiconductor (MOS) memory with Quattro-layer graphene-nanoplatelets as charge storage layer with asymmetric Al_2_O_3_/HfO_2_ tunnel oxide and we compare it to the same memory structure with 2.85-nm Si nanoparticles charge trapping layer. TEM images, electrical characterization, construction of the energy band diagrams of the MOS memory devices, and quantum mechanical calculations are provided to confirm the importance of the band-engineering of both tunnel oxide and charge trapping layer of non-volatile memory devices. In addition, the results show that MOS memory devices with Quattro-layer graphene-nanoplatelets as charge storage layer with asymmetric Al_2_O_3_/HfO_2_ tunnel oxide has potential in future low-power and fast non-volatile memory devices.

## Methods

**Fig. 1:**
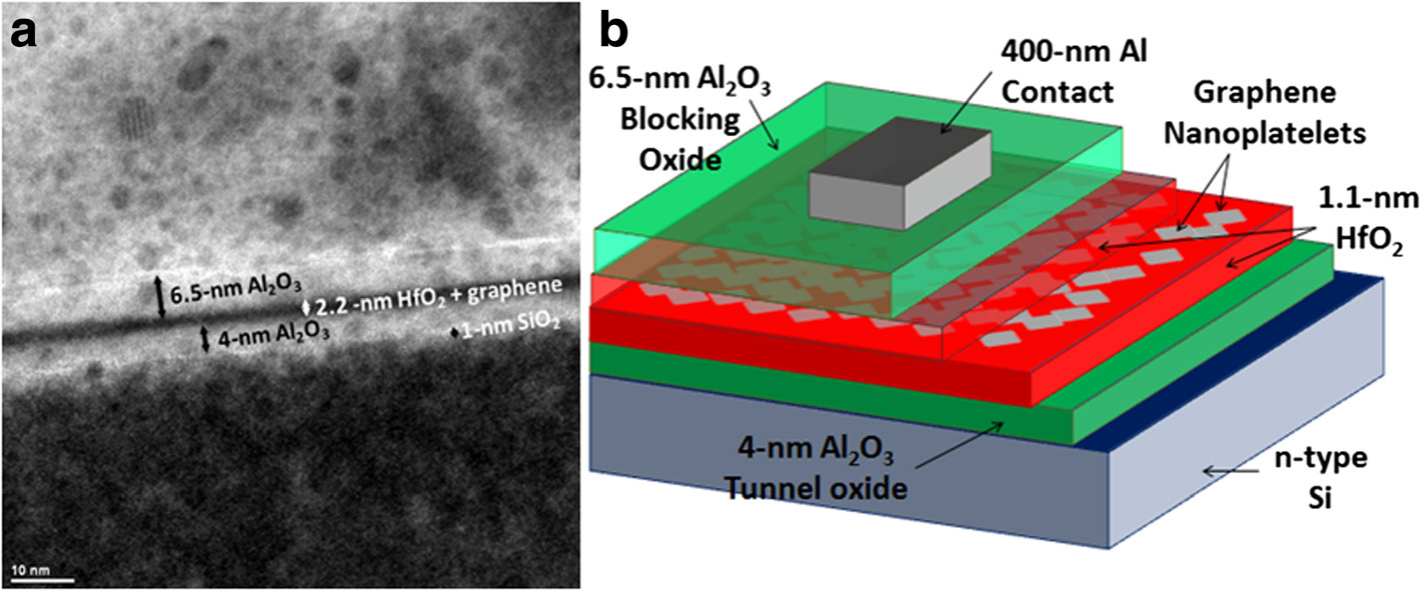
Fabricated memory devices; **a** TEM cross-section of the memory with graphene nanoplatelets. **b** Cross-section illustration of the fabricated memory cells with graphene nanoplatelets. The memory with Si nanoparticles has the same cross-section illustration

The MOS memory devices are fabricated on low-resistivity n-type Si(111) substrate (Antimony-doped, 15–20 mΩ/cm). A 4-nm Al_2_O_3_ tunnel oxide is first deposited by thermal atomic layer deposition (ALD) at 250 **°**C using a Cambridge Nanotech Savannah-100 atomic layer deposition system followed by 1.1 nm HfO_2_ deposited by plasma-assisted ALD (PA-ALD) at 195 **°**C using an Oxford FlexAL system. Next, the sample is placed on a hot plate at 110 **°**C, and 2–2.5 ml of pristine graphene nanoplatelets (Quattro-layer, 0.05 mg/ml) with an average size of 4.4 nm (see Additional file 1: Figure S1) are drop-casted on the sample. Then, 1.1 nm HfO_2_ is deposited by PA-ALD at 195 **°**C followed by 6.5-nm Al_2_O_3_ blocking oxide deposited at 250 **°**C by ALD. Finally, a shadow mask with feature size down to 10 μm is used to pattern the 400-nm Al gate contact deposited by e-beam evaporation. The same process is repeated to fabricate the MOS memory with 2.85-nm Si nanoparticles [7] (see Additional file 1: Figure S2), where Si nanoparticles are spin-coated on the sample at a speed of 2000 rpm and acceleration of 500 rpm/s for 45 s. TEM cross-section of the MOS memory with graphene nanoplatelets is shown in Fig. 1a where an interfacial 1 nm SiO_2_ is observed (see Additional file 1: Figure S3 also). A cross-section illustration of the fabricated memory with graphene nanoplatelets is also shown in Fig. 1b.

The electrical measurements are done using an Agilent B1505A semiconductor device analyzer.

## Results and Discussion

**Fig. 2:**
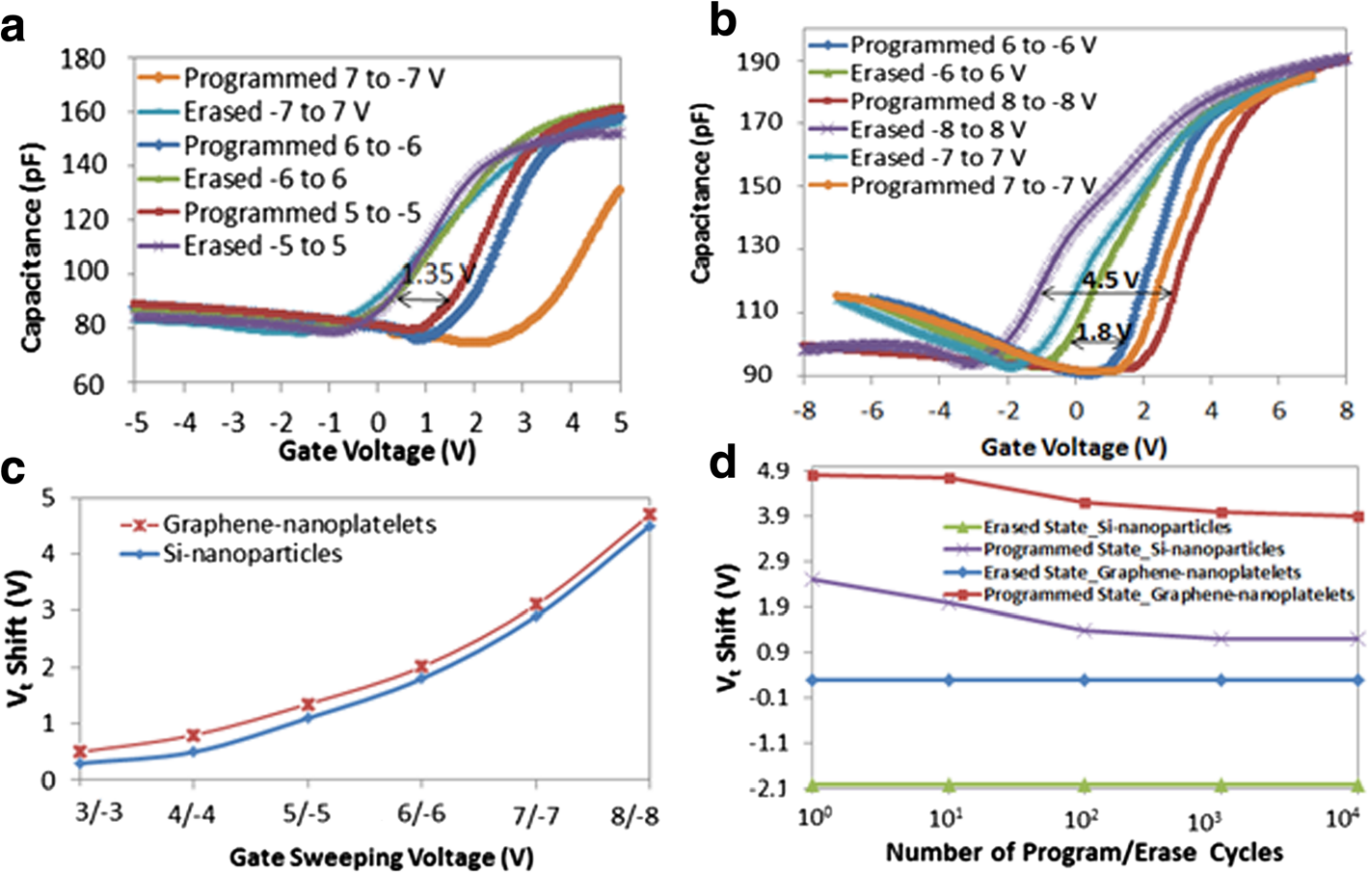
Electrical characterization of the memory devices; **a** High-frequency (1 MHz) C-V measurements of the memory with graphene nanoplatelets. **b** High-frequency (1 MHz) C-V measurements of the memory with Si nanoparticles. **c** Plot showing the measured *V*_t_ shifts at different gate sweeping voltages. **d** Endurance characteristic of the memory devices programmed/erased at 8/−8 V at room temperature

To analyze the memory performance, high-frequency (1 MHz) C-V_gate_ measurements are conducted. The gate voltage is first swept from −7 to 7 V which resulted in the erased-state, then from 7 to −7 V resulting in the programmed state. The obtained memory hysteresis is 3.1 V with graphene nanoplatelets while 2.9 V with Si nanoparticles. The measurements are repeated at different gate voltages as shown in Fig. 2a, b for the memory with graphene nanoplatelets and Si nanoparticles, respectively. It is observed that the memory with Si nanoparticles is programmed by storing electrons and erased by storing holes as shown by the positive and negative shifts in the programmed and erased states of Fig. 2b, respectively. It is also shown in Fig. 2b that additional charging is due to holes at large erasing voltages of −8 V corresponding to an electric field across the tunnel oxide Al_2_O_3_ (*E*_ox_) of 10.6 MV/cm whereas the memory with graphene nanoplatelets is programmed by storing electrons and erased through back-tunneling of electrons which is shown by the shift of the programmed state in Fig. 2a. The threshold voltage (*V*_t_) shift achieved with graphene nanoplatelets is higher than the *V*_t_ shift achieved with Si nanoparticles at different gate voltages as shown in Fig. 2c.

The memory endurance characteristic is studied by plotting the *V*_t_ shift vs*.* the number of program/erase cycles at 8/−8 V as depicted in Fig. 2d. Non-volatile memories can be programmed/erased frequently at the expense of introducing permanent gate-oxide damage such as the trapping of electrons/holes in the available trapping states in the oxide [8]. These trapped charges change the injection fields and, thus, the amount of charge transferred to and from the charge storage layer during programming. The lower endurance with Si nanoparticles after 10^4^ cycles (33.3 % degradation) than the memory endurance with graphene (20 %) can be due to two reasons: first, the larger accumulation capacitance (*C*_acc_) of the memory with Si nanoparticles and the similar Δ*V*_t_ at 8 V results in a larger trapped charge density (Δ*Q* = *C*_acc_ × Δ*V*_t_) in the Si nanoparticles (ΔQ in Si nanoparticles ~8.3 × 10^13^ cm^−2^>Δ*Q* in graphene nanoplatelets ~7.3 × 10^13^ cm^−2^) which means that more charges are tunneling through the tunnel oxide of the memory with Si nanoparticles which might increase the degradation of the oxide. Second, with Si nanoparticles, both electrons and holes are tunneling through the tunnel oxide during program/erase cycles. As a result, both electrons and holes will be trapped in the available trapping states in the oxide further degrading the endurance characteristic with respect to the memory with graphene nanoplatelets where only electrons are tunneling.

**Fig. 3:**
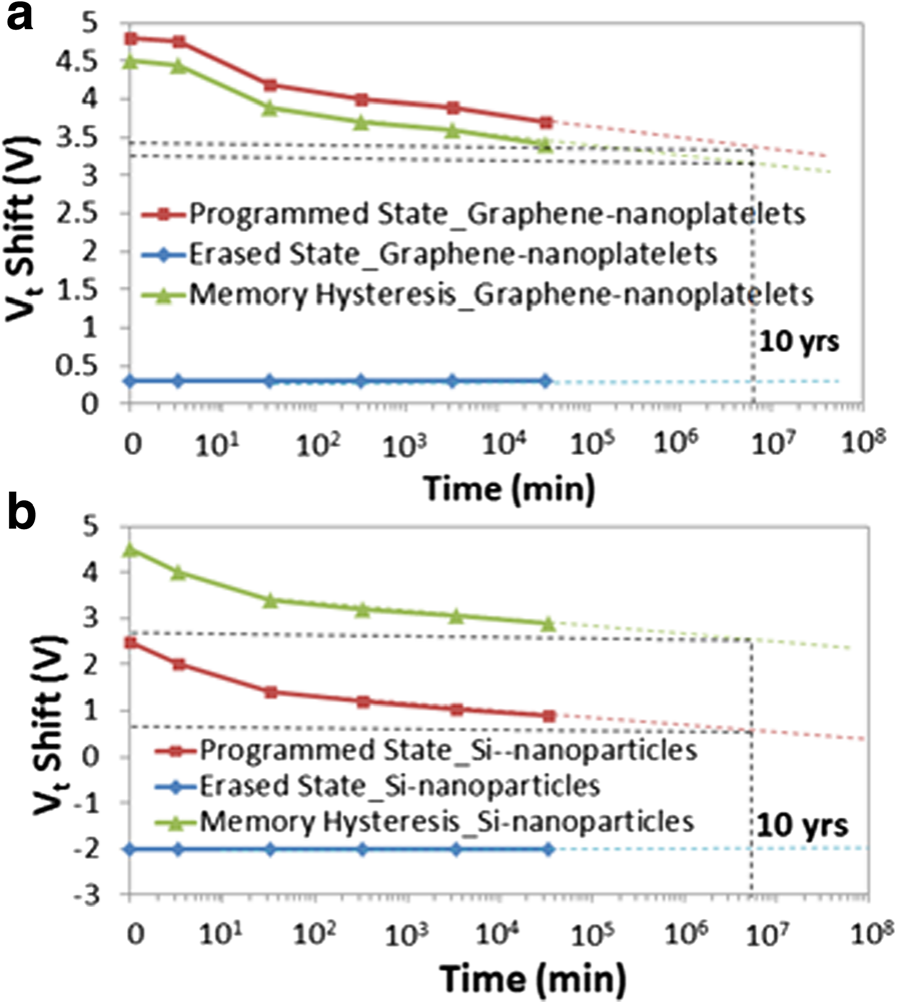
Memory retention characteristics measured by first programming/erasing the memory at 8/−8 V at room temperature **a** with graphene nanoplatelets and **b** with Si nanoparticles

Moreover, the retention of the memory cells is characterized by first programming/erasing the memory at 8/−8 V and observing the change in *V*_t_ shift in time as shown in Fig. 3a, b for the memory devices with graphene nanoplatelets and Si nanoparticles, respectively. The enhanced retention with graphene (28.8 % loss of initial stored charge) at 10 years with respect to the retention of the memory with Si nanoparticles (35.5 %) is due to the larger electron affinity of graphene [9] (4.6 eV) than 2.85-nm Si nanoparticles [10] (2.9 eV) which increases the conduction band offset (CBO) between charge storage layer and tunnel oxide, and therefore exponentially reduces the back-tunneling of electrons.

**Fig. 4:**
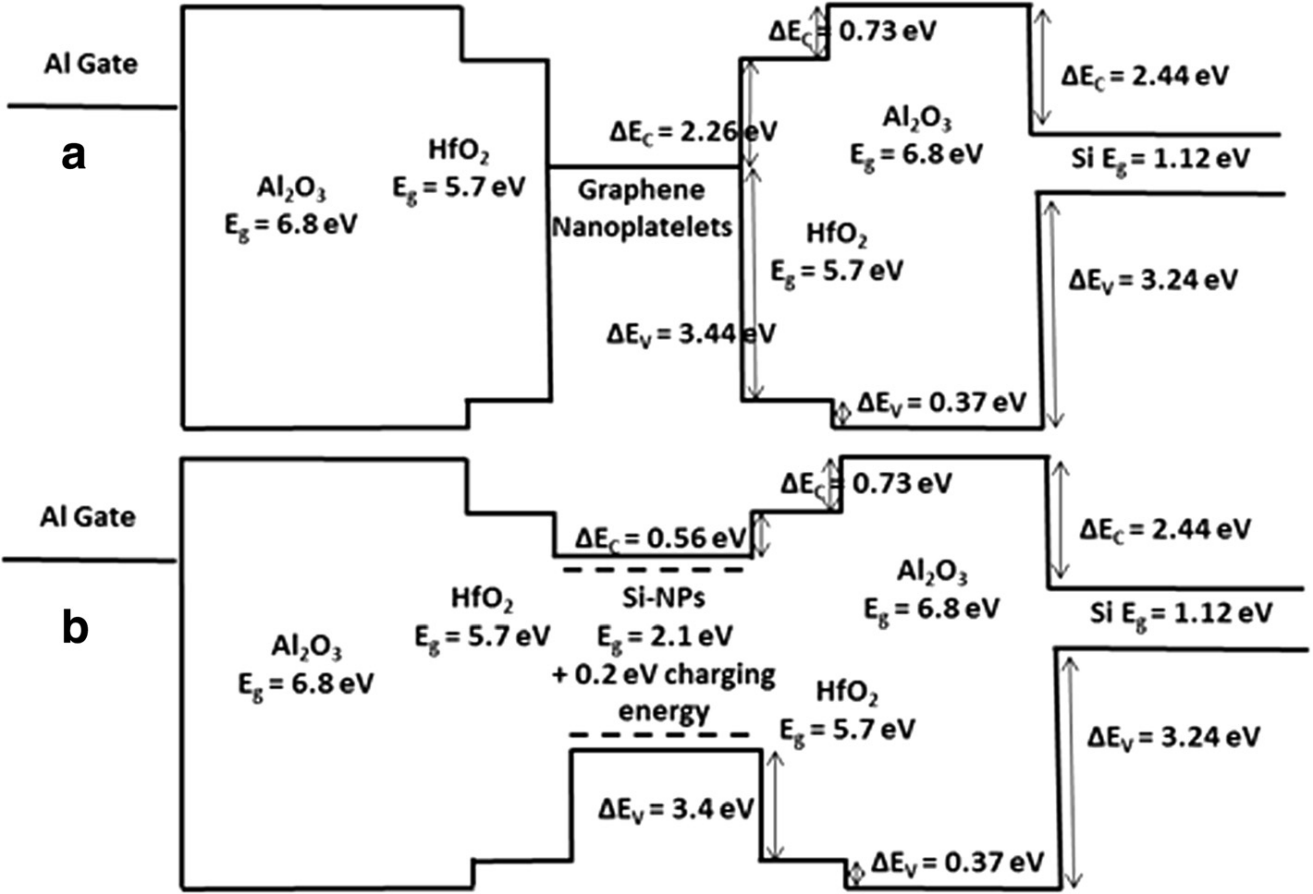
Energy band diagram of the memory **a** with graphene nanoplatelets and **b** with Si nanoparticles. The energy band diagram of the memory with Si nanoparticles takes into consideration the changes due to quantization and coulomb charging energy of the 2.85 nm Si nanoparticles

**Fig. 5:**
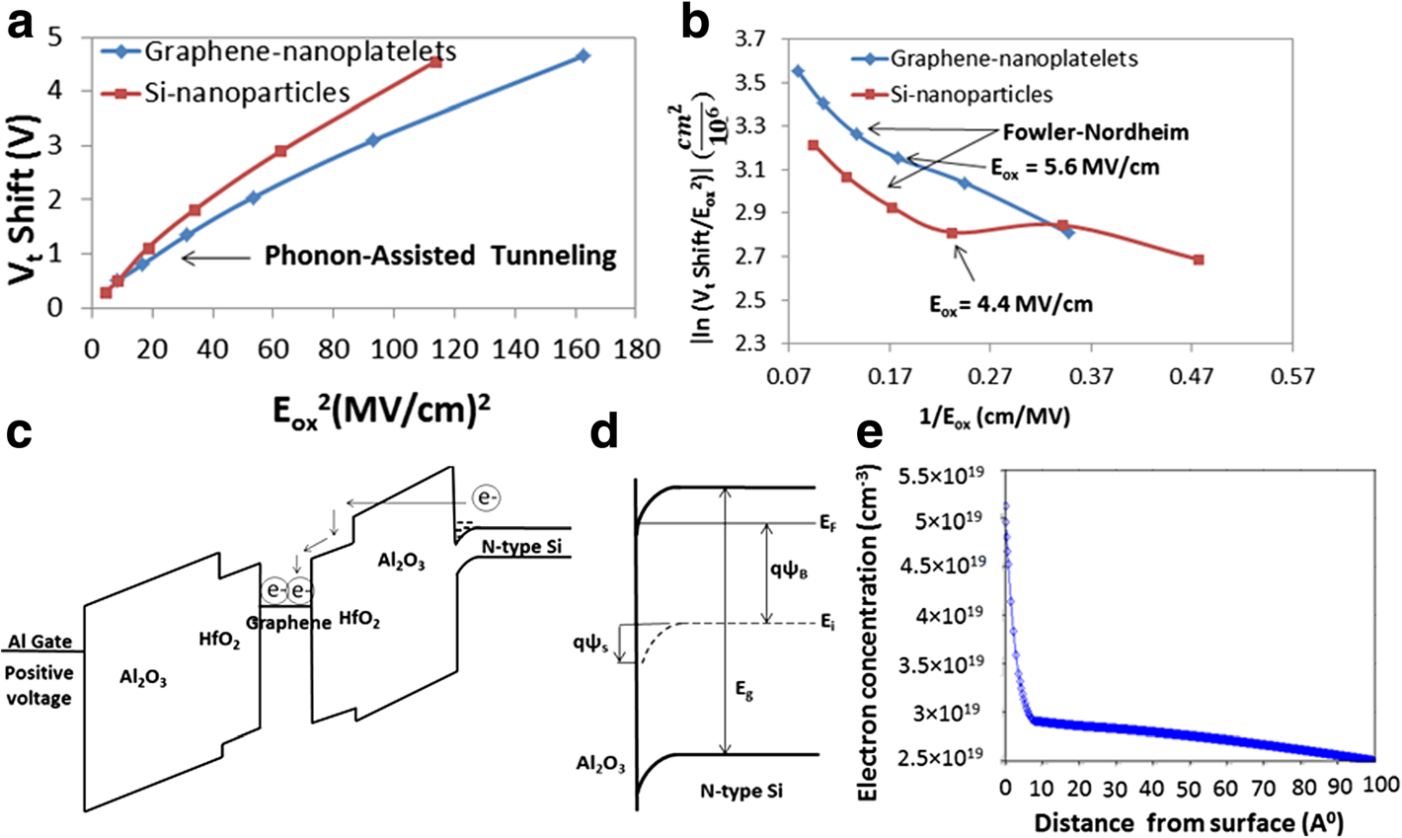
Charge transport mechanism; **a** Plot showing the *V*_t_ shift vs*.* the square of the electric field across the Al_2_O_3_ for both memories. **b** Plot showing the natural logarithm of the *V*_t_ shift divided by the square of the electric field vs*.* the reciprocal of the electric field across Al_2_O_3_. **c** Energy band diagram of the memory with graphene nanoplatelets under positive gate voltage. **d** Energy band diagram near the Si interface of the memory with graphene nanoplatelets. **e** Plot showing the accumulation electron charge density vs*.* the distance from the Si interface

The energy band diagrams of the memory structures with graphene and Si nanoparticles are plotted in Fig. 4a, b, respectively [11–22]. The smaller CBO than valence band offset (VBO) between the substrate and Al_2_O_3_ confirms the observed electrons storage during programming of both memories. In order to analyze the charge emission mechanism, the electric field across Al_2_O_3_ is calculated using Gauss’s law [17], and the *V*_t_ shift vs*.* (*E*_ox_)^2^ is plotted in Fig. 5a, and the linear region suggests that phonon-assisted tunneling (PAT) [17] is the main emission mechanism at *E*_ox_ < 5.6 MV/cm. The plot of the natural logarithm of the *V*_t_ shift divided by the square of the electric field vs*.* the reciprocal of the electric field ($J={C}_1{E}_{{\mathrm{ox}}^2}^2{e}^{-\frac{C_2}{E_{\mathrm{ox}}}}$) depicted in Fig. 5b shows a linear region at *E*_ox_>5.6 MeV/cm confirming that Fowler-Nordheim tunneling [17] becomes dominant at higher electric fields. In this case, electrons tunnel through the Al_2_O_3_ triangular energy barrier and are swept by the electric field into the conduction band of HfO_2_ then into the conduction band of the graphene nanoplatelets as shown in Fig. 5c.

Also, the larger CBO between graphene and Al_2_O_3_ compared to the CBO between Si nanoparticles and Al_2_O_3_ confirms the enhanced retention with graphene. The trap lifetime of the electrons and holes in the memory devices is calculated by first finding the back-tunneling probability (*T*) [17, 23]:

1 $$ \mathrm{T}=16\times \left(\frac{E_0}{V_0}\right)\times \left(1-\frac{E_0}{V_0}\right)\times {e}^{-2d\ \frac{\sqrt{2{m}_0\left({V}_0-{E}_0\ \right)}}{\hslash }} $$

where *V*_0_ is the potential energy of the barrier, *d* is the thickness of the barrier, *m*_0_ is the effective mass in the oxide, and *E*_0_ is the ground state energy of the electron trapped in a 4.4-nm quantum well (in the case of the graphene nanoplatelets) and is equal to ${E}_0=\frac{\hslash^2{\pi}^2}{2{m}_0{L}^2}$ where *ћ* is the reduced Plank’s constant and *L* is the thickness of the storage layer [24–34]. Since in the demonstrated memory devices there are three barriers (HfO_2_, Al_2_O_3_, and interfacial SiO_2_) that the electron must tunnel through to leak-out, the total transmission probability is thus found by multiplying the transmission probabilities through each oxide and total *T* is found equal to ~2 × 10^−23^ for the memory with graphene nanoplatelets. The electron trap lifetime can be then estimated by *τ*_e_ = (*υT*)^−1^ = 7.14 × 10^8^ s~23.7 years where the attempt frequency *υ* in a quantum well [25] is $\frac{E_0}{2\pi \hslash }=7\times {10}^{13}{\mathrm{s}}^{-1}$. Similar calculations are performed for the case of the memory with Si nanoparticles, and the electron trap lifetime is found to be *τ*_e_~15.7 years while the holes trap lifetime is τ_h_~30 years which is expected to be much larger due to the very large VBO between Si nanoparticles and Al_2_O_3_ (Δ*E*_V_ = 3.81 eV). The calculated results support the measured memory retention characteristic.

Furthermore, the program times for both memories are calculated. Since during the program operation, the electron tunnels through Al_2_O_3_ by Fowler-Nordheim tunneling and is swept by the electric field to the charge trapping layer, then the program speed can be found by multiplying the probability of Fowler-Nordheim tunneling through the Al_2_O_3_ layer (*T*_FN_) by the attempt-to-escape frequency (*υ*_p_). *T*_FN_ can be estimated from Eq. (2) [25, 26]:

2 $$ {T}_{\mathrm{FN}}={e}^{-\frac{4}{3}\frac{\sqrt{2{m}_0}}{h}\frac{f^{\frac{3}{2}}}{e{E}_{\mathrm{ox}}}} $$

where *Φ* is the CBO between substrate and Al_2_O_3_, *E*_ox_ is the electric field across Al_2_O_3_, and *e* is the elementary charge. Since during the program operation, there will be band-bending of the Si substrate near the interface with Al_2_O_3_, a triangular barrier is formed as shown in Fig. 5c, d, and the attempt-to-escape frequency in a triangular barrier is [26]:

3 $$ {\upsilon}_p=\sqrt{\frac{2{E}_1q}{m_0}}\frac{1}{2w} $$

where ${E}_1=2.34\times {\left\{\left[\frac{{\left(q{E}_{\mathrm{ox}}\hslash \right)}^2}{2{m}_0}\right]\right\}}^{\frac{2}{3}}$ and *w* is the thickness of the triangular barrier which can be estimated very well by the accumulation region thickness. The electron concentration in the substrate during accumulation is plotted vs*.* the distance from surface as shown in Fig. 5e. At a program voltage of 8 V, the charge density in the accumulation region can be estimated from [17] *Q* = (*V*_p_–*V*_t_) × *C*_i_ where *V*_p_ is the program voltage and *C*_i_ is the oxide capacitance per unit area. The corresponding volume charge density is *Q*_acc_ = 3.05 × 10^19^ cm^–3^ with graphene nanoplatelets which corresponds to an accumulation region thickness of *w* = 6 A^0^ as shown in Fig. 5e. Therefore, the program time is calculated by dividing the stored charge *Q* given by $Q=\frac{V_{\mathrm{t}\ \mathrm{shift}}}{q\times {C}_{\mathrm{i}}}$ where *C*i is the oxide capacitance, by the program speed, and it is found to be equal to 4.1 ns at 8 V with graphene nanoplatelets which is much faster than reported non-volatile memory program times in literature (32 ns at 12 V [34], 100 ns at 10 V [35], 1 μs at 10 V [36]). With Si nanoparticles, the time needed for the electrons to tunnel through Al_2_O_3_ is similarly calculated and found 5.6 ns which is larger than the write time of the memory with graphene nanoplatelets mainly due to the lower electric field across the tunnel oxide in the memory with Si nanoparticles. However, in the case of Si nanoparticles, the time needed to program the memory is found by adding the time needed for the holes to tunnel back to the substrate as well (since mixed charging is observed in this memory) which results in a program time >>5.6 ns.

## Conclusions

In conclusion, memory devices with Quattro-layer graphene nanoplatelets and 2.85-nm Si nanoparticles with Al_2_O_3_/HfO_2_ tunnel oxide are demonstrated. The results show that graphene nanoplatelets provide a larger charge trapping state density revealed by the larger memory window, enhanced memory endurance due to the pure electrons storage, and enhanced retention due to the larger conduction band offset between storage layer and Al_2_O_3_. Also, the graphene nanoplatelet memory showed a faster program speed compared to Si nanoparticle memory. Finally, the results confirm that band-engineering of both tunnel oxide and charge trapping layer is essential to enhance the memory characteristics. Also, the results highlight that such memory structures have potential in next-generation non-volatile memory devices.

## Additional file

Additional file 1:
**Supplementary information.** The supplemental information include an AFM image of the graphene nanoplatelets, a TEM image of the Si nanoparticles, and a high-angle annular dark-field (HAADF) STEM image of the cross-section of the memory with graphene, in addition to calculations of the accumulation charge concentrations.
