# Supplementary material for: Enhanced non-volatile memory characteristics with quattro-layer graphene nanoplatelets vs. 2.85-nm Si nanoparticles with asymmetric Al2O3/HfO2 tunnel oxide
Source: Nanoscale Res Lett. 2015 Jun 2;10:248. doi: 10.1186/s11671-015-0957-5 (PMC4456595; doi:10.1186/s11671-015-0957-5)
Supplement: Additional file 1: — Supplementary information. The supplemental information include an AFM image of the graphene nanoplatelets, a TEM image of the Si nanoparticles, and a high-angle annular dark-field (HAADF) STEM image of the cross-section of the memory with graphene, in addition to calculations of the accumulation charge concentrations. [file 11671_2015_957_MOESM1_ESM.pdf]

Supplementary Information

By Nazek El-Atab, Berk Berkan Turgut, Ali K. Okyay, Munir Nayfeh, and Ammar Nayfeh

Graphene-nanoplatelets: The used graphene solution with concentration 0.05 mg/mL is comprised entirely of pristine graphene-nanoplatelets which have not been oxidized, reduced or chemically modified. The average size of the Quattro-layer graphene nanoplatelets is 4.4-nm (each layer is 1.1 nm). Fig.1 shows the AFM image of the very small graphene nanoplatelets dispersed on an SiO_2 substrate.

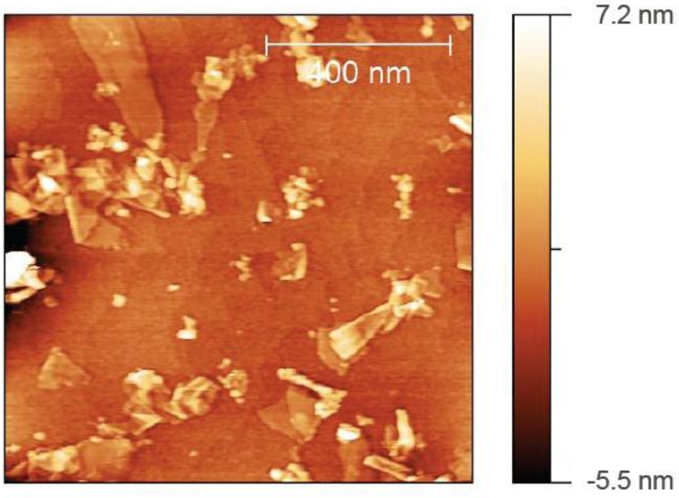

Figure 1: AFM image of several pristine graphene-nanoplatelets on an SiO_2 substrate. (From NanoIntegris PureSheets Quattro datasheet).

Si-nanoparticles synthesis: The Si-NPs used in this study were supplied by NanoSi Advanced Technology Inc. The nanoparticles were prepared from Si wafers by chemical etching in HF and H_2O_2 using electrical or hexachloroplatinic acid catalyst [1-4]. After the treatments the wafers were sonicated in a liquid of choice and form a colloid that is stable over many years. The 2.85-nm average size particles were produced with a highly stable hydrogen termination [5]. Figure 2.a gives a prototype of the smallest particle which is

$Si_{29}H_{24}$ with 5 atoms constituting a tetrahedral core and 24 atoms constituting an H-terminated reconstructed Si surface. A single H atom terminates each surface Si atom. Fig. 2b shows the TEM image of the ultra-small non-agglomerate Si-nanoparticles.

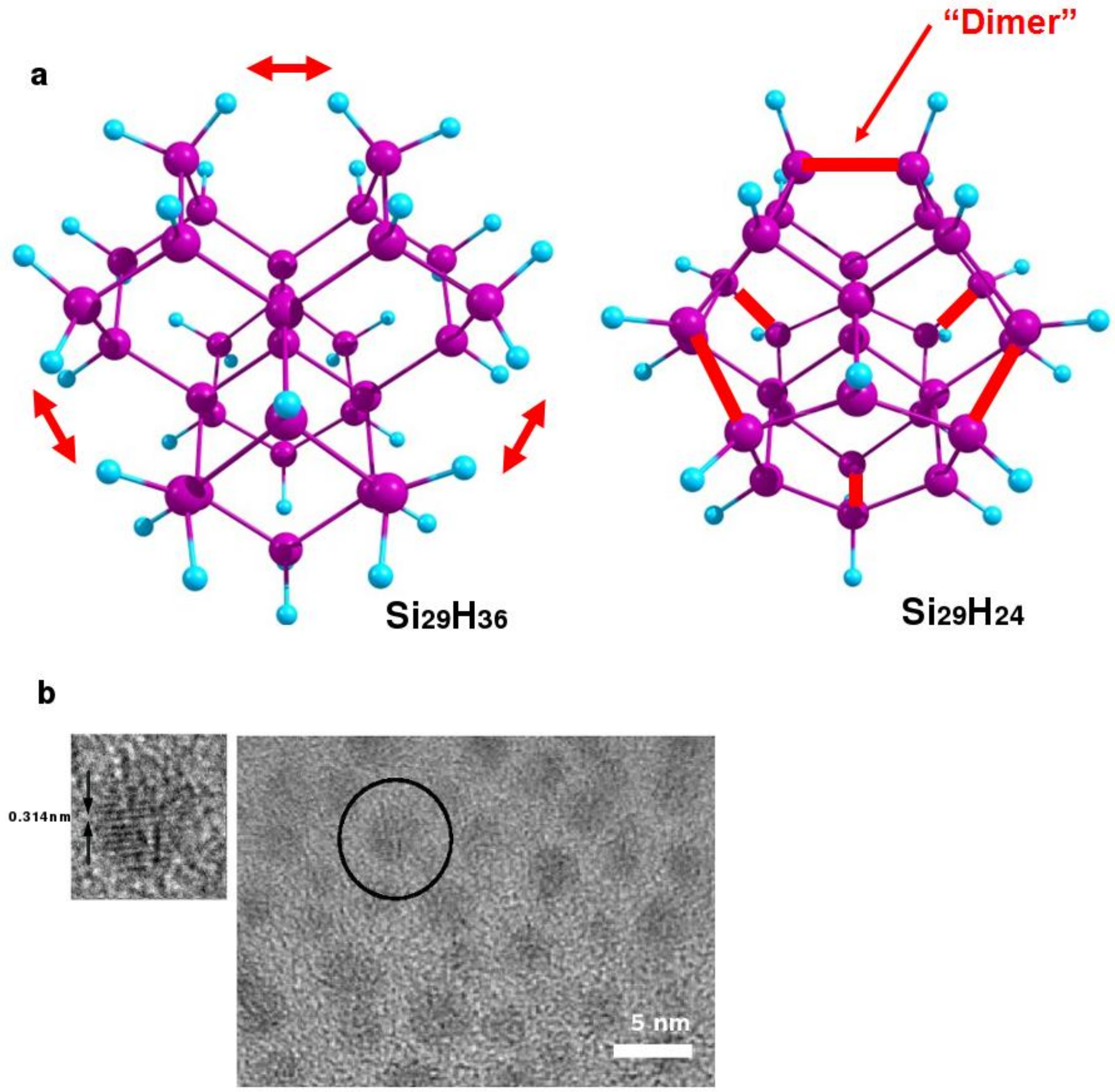

Figure 2. a) Structures of $Si_{29}H_{36}$ and $Si_{29}H_{24}$. In the $Si_{29}H_{24}$ particle, 5 Si atoms (purple circle) constitute a single tetrahedral core and 24 Si atoms (purple circle) constitute a H-terminated (blue circle) reconstructed surface b) TEM image of showing ultra-small and non-agglomerate Si nanoparticles.

TEM of the memory with graphene-nanoplatelets: Samples were preprocessed carefully with a focused ion beam (FIB) tool before imaging in TEM. Owing to relatively high energy levels of TEM tool, the graphene layer gets deteriorated easily. So it is quite difficult to

image the graphene flakes directly. We used HAADF STEM technique that provides a contrast between species with different atomic numbers. An annular dark field image formed only by very high angle, incoherently scattered electrons — as opposed to Bragg scattered electrons — is highly sensitive to variations in the atomic number of atoms in the sample (Z-contrast images). This technique is also known as high-angle annular dark-field imaging (HAADF). The dark regions in Fig. 3a show 4 nm and 6.5 thick Al2O3 layers on Silicon (100) substrate whereas the white strip shows graphene and HfO2 layers. Fig. 3b shows the HAADF intensity signal profile along the green line corresponding to the trap region as shown in Fig. 3a. The plato region in this signal profile exhibits a dip in the signal intensity. This dip corresponds to graphene flakes, which correspond to dark spots in the bright strip in Fig. 3a. For a pure HfO2 film, the intensity profile would be flat with no dips.

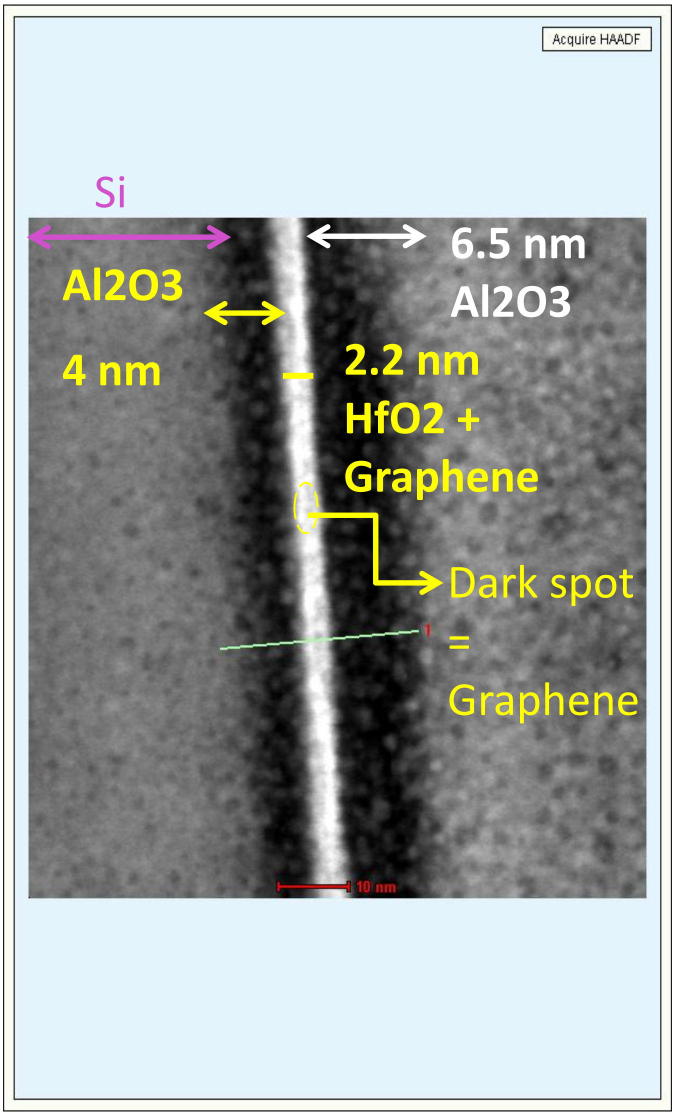

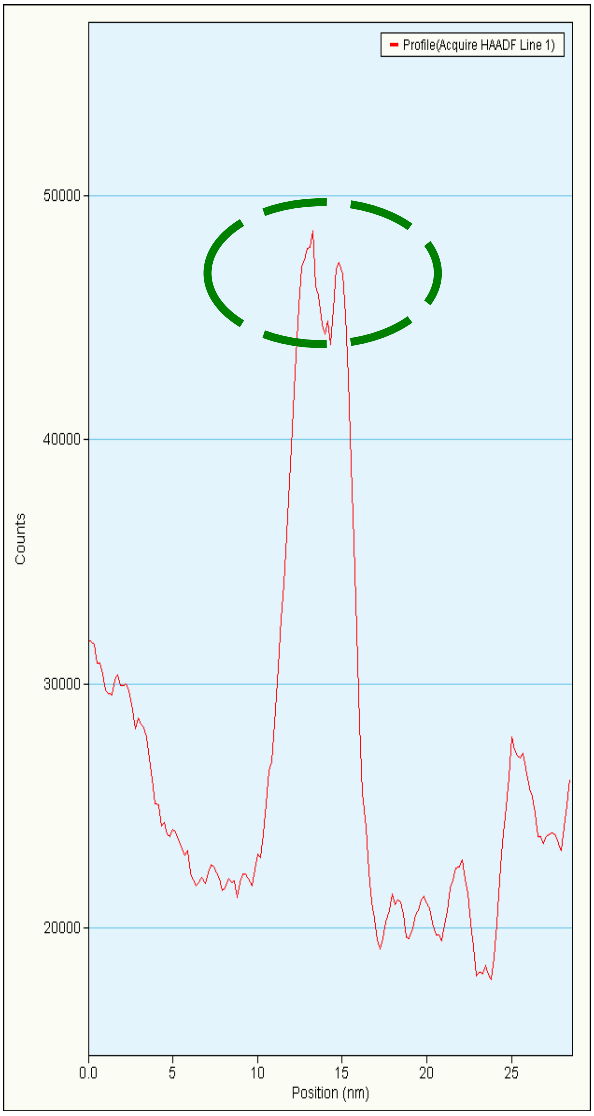

Figure 3a **Figure 3b**

Figure 3: a) HAADF STEM image of the cross-section of the memory with graphene-nanoplatelets. b) HAADF intensity signal profile along the green line corresponding to the trap region as shown in Fig. 3a.

Substrate Doping Concentration: The n^+-type substrates (Antimony doped) have a resistivity of 15-20 mΩ/cm. However, the exact doping concentration is calculated from the C-V characteristics. The depletion capacitance C_d is first calculated using equation (1):

$$\frac{1}{C_{min}} = \frac{1}{C_i} + \frac{1}{C_d} \tag{1}$$

where C_{min} is the minimum capacitance and C_i is the oxide capacitance. C_d is found equal to 211.11 pF. The depletion layer thickness (W) is then calculated using equation (2):

$$C_{d/A} = \frac{\epsilon_s}{W} \tag{2}$$

where $C_{d/A}$ is the depletion capacitance per unit area and ϵ_s is the dielectric constant of Si. Therefore, W is found equal to 9.86×10^{-7} cm. Using this value, the substrate doping N_D can be calculated recursively using equation (3):

$$W = \left[\frac{4\epsilon_S \frac{KT}{q} ln \frac{N_D}{n_i}}{qN_D}\right]^{\frac{1}{2}} \tag{3}$$

where n_i is the intrinsic concentration of silicon and KT/q is the thermal voltage. N_D is found equal to 1.45×10^{19} cm^{-3}.

Accumulation Charge Concentration: The charge concentration plot vs. distance from surface is obtained by first solving the differential equation of the band bending ψ with respect to the distance from the Si-surface given in equation (4):

$$\frac{d\psi}{dx} = -\sqrt{\frac{2KTN_D}{\epsilon_{Si}}\left(\frac{q\psi}{KT} + \frac{n_i^2}{N_D^2} e^{\frac{q\psi}{KT}}\right)} \tag{4}$$

The boundary condition is $\psi=\psi_s$ at x=0 where ψ_s is the band bending at strong inversion, therefore $\psi_s = 0.5E_g - \psi_B$ and ψ_B is the difference between the Fermi level E_F and the intrinsic level E_i, thus $\psi_B = \frac{KT}{q} ln \frac{N_D}{n_i}$.

After ψ(x) is solved, the electrons distribution $n(x)$ in the accumulation layer can be

calculated using equation (5):

$$n(x) = N_D e^{\frac{q\psi(x)}{KT}} + N_D \quad (5)$$

The electron concentration in the accumulation layer is then plotted by plotting *n(x)* vs. *x*.

[1] O. Ackakir, J. Therrien, G. Belomoin, N. Barry, J. Muller, E. Gratton, and M. Nayfeh, "Detection of luminescent single ultrasmall silicon nanoparticle using fluctuation spectroscopy," *Applied Physics Letters,* vol. 76, pp. 1857-1859, 2000.

[2] G. Belomoin, J. Therrien, A. Smith, S. Rao, S. Chaieb, M. H. Nayfeh, "Observation of a magic discrete family of ultrabright Si nanoparticles," *Applied Physics Letters,* vol. 80, pp. 841, 2002.

[3] D. Nielsen, L. Abuhassan, M. Alchihabi, A. Al-Muhanna, Jon Host, and M. H. Nayfeh, "Current-less anodization of intrinsic silicon powder grains: Formation of fluorescent Si nanoparticles," *Journal of Applied Physics*, vol. 101, pp. 114302, 2007.

[4] M. H. Nayfeh, and L. Mitas, " Silicon nanoparticles: new photonic and electronic material at the transition between solid and molecule," *Nanosilicon*, Ch. 1, pp. 3-6, 2007.

[5] Z. Yamani, H. Thompson, L. AbuHassan, and M.H. Nayfeh, " Ideal anodization of silicon," *Applied Physics Letters,* vol. 70, pp. 3404, 1997.
